# Virtual Photovoice With Older Adults: Methodological Reflections during the COVID-19 Pandemic

**DOI:** 10.1177/16094069221095656

**Published:** 2022-05-07

**Authors:** Olivier Ferlatte, Julie Karmann, Geneviève Gariépy, Katherine L. Frohlich, Gregory Moullec, Valérie Lemieux, Réjean Hébert

**Affiliations:** 1 École de Santé Publique, 5622 Université de Montréal (ESPUM), Montreal, QC, Canada; 2 Centre de Recherche en Santé Publique (CReSP), Université de Montréal et CIUSSS du Centre-Sud-de-l’Île-de-Montréal, Montreal, QC, Canada; 3 CIUSSS du Nord-de-l’Île-de-Montréal, Montreal, QC, Canada; 4 CIUSSS du Centre-Sud-de-l’Île-de-Montréal, Direction Régionale de Santé Publique de Montréal, Montreal, QC, Canada

**Keywords:** photovoice, older adults, Covid-19, online research, online study, mental health

## Abstract

Photovoice is a participatory action research method in which participants take and narrate photographs to share their experiences and perspectives. This method is gaining in popularity among health researchers. Few studies, however, have described virtual photovoice data collection despite the growing interest among qualitative health researchers for online data collection. As such, the aim of this article is to discuss the implementation of a virtual photovoice study and presents some of the challenges of this design and potential solutions. The study examined issues of social isolation and mental health among older adults during the COVID-19 pandemic in the Canadian province of Québec. Twenty-six older adults took photographs depicting their experience of the pandemic that were then shared in virtual discussion groups. In this article, we discuss three key challenges arising from our study and how we navigated them. First, we offer insights into managing some of the technical difficulties related to using online meeting technologies. Second, we describe the adjustments we made during our study to foster and maintain positive group dynamics. Third, we share our insights into the process of building and maintaining trust between both researchers and participants, and amongst participants. Through a discussion of these challenges, we offer suggestions to guide the work of health promotion researchers wishing to conduct virtual photovoice studies, including with older adults.

## Background

Photovoice is gaining in popularity as a qualitative and participatory research method (Golden, 2020). Few studies, however, have described the use of virtual photovoice despite a growing interest among qualitative researchers in doing internet-based data collection (Fielding et al., 2016). Online research designs may be useful to overcome some of the barriers inherent to qualitative research design by offering greater flexibility in time and location of data collection. Online research can also allow for participation across a wide geographic area, providing for greater diversity (Fielding et al., 2016; Janghorban et al., 2014; Reisner et al., 2018). Yet, conducting data collection online presents some challenges specific to photovoice that have yet to be detailed in the literature. To address this gap, this article presents our experience conducting a photovoice study with older adults entirely online during the COVID-19 pandemic and explores some of the challenges with this method when used with this population.

### Photovoice

Initially developed as a research method to gain knowledge of Chinese women’s experiences (Wang & Burris, 1997), photovoice is a qualitative research method that combines photo taking and interviews/focus groups, and/or writing about the meaning and content of the images. Rooted in the principles of participatory action research, its goal is to empower communities to document and communicate their lived experience to drive social change (Hergenrather et al., 2009). Photovoice is based on the assumptions that participants are experts about their health and the social issues they face and should therefore be involved in the production of knowledge about them (Catalani & Minkler, 2010).

Since its inception in the late 1990s (Wang & Burris, 1997), photovoice is increasingly becoming popular to study issues related to health promotion and health inequities (Catalani & Minkler, 2010; Golden, 2020). This growing interest is driven in part by the numerous benefits of this method that have been detailed in its literature. Photovoice can increase accessibility and facilitate the investigation of sensitive and complex issues by offering an alternative to direct interview questions (Catalani & Minkler, 2010). Photographs can provide a buffer to the potential awkwardness invoked by structured interviews that can feel like an examination (Oliffe & Bottorff, 2007). Because the conversation begins with the participants photographing what they believe is most important about their experience, it shifts authority from the researcher to the research participants and ensures that the research reflects the priorities of affected communities (Ferlatte & Oliffe, 2019b; Oliffe & Bottorff, 2007). Finally, it offers innovative community-based knowledge translation opportunities that can help advocate for policy change (Ferlatte & Oliffe, 2019a).

Key to photovoice method is the idea of empowerment (Liebenberg, 2018; Wang & Burris, 1997). Through the process of participating in a photovoice project, participants acquire new knowledge and skills, and can develop a critical awareness of an issue, their own experiences or of their community (Han & Oliffe, 2016; Sitvast et al., 2010). If done as a group, photovoice can empower participants by allowing them to expand their social networks and to build new links with various actors, including researchers (Budig et al., 2018; Duffy, 2011). When photographs are disseminated to the public and policy-makers to raise awareness of a given issue or to promote social change, participants can develop a sense of pride of having voiced their perception and experience, and see their self-perception transformed (Ferlatte & Oliffe, 2019a; Strack et al., 2004).

Photovoice has been used with diverse populations across the life course including children, youth, adults (Catalani & Minkler, 2010; Golden, 2020), and, to a much lesser extent, older adults (Novek et al., 2012; Bryanton et al., 2019). While use of photovoice with older adult populations is still limited, it nonetheless offers an effective and novel way to engage this population which is often excluded from research and other aspects of social life. Previous studies have used photovoice to produce knowledge on older adults and issues of health promotion such as mental health (Panazzola & Leipert, 2013), diabetes (Yankeelov et al., 2015), age-friendly environments (Novek & Menec, 2014), physical activity (Mahmood et al., 2012), chronic pain (Baker & Wang, 2006) and cardiovascular diseases (Fitzpatrick et al., 2012). Researchers have, however, noted some important challenges to the implementation of photovoice studies with older adults. Specifically, mobility and vision problems, not uncommon among older adults, may limit the ability of some older adults to manipulate a camera or to reach places they would consider meaningful to photograph for the research question (Novek et al., 2012). Older adults may also lack familiarity with certain new technologies and camera use and as such be reluctant to participate in photovoice studies (Novek et al., 2012).

While the photovoice method is gaining in popularity, very few studies have described photovoice data collection undertaken completely virtually (Lichty et al., 2019; Tanhan & Strack, 2020), and none to our knowledge among older adults. Because of their lack of in-person interactions, online studies can lead to participants feeling less engaged and expressing fewer nonverbal signals, which may lead to participants misunderstanding each other or cause researchers to miss important nuances (Reisner et al., 2018). With older adults, these challenges may be compounded by a lack of skills and confidence in using new technologies (Hunsaker & Hargittai, 2018) and may create or exacerbate a reticence to engage in online research. More research is therefore needed to examine how photovoice could be successfully implemented online in general, and with older adults in particular.

### Confinés, Ensemble!: A Virtual Photovoice Project during the COVID-19 Pandemic

Faced with the threat of COVID-19, the provincial government of Quebec, Canada, declared a health state of emergency on March 13^th^, 2020, and swiftly implemented a series of measures to limit the spread of the virus, including lockdown measures and physical distancing, and recommended that older adults stay home unless necessary because of their increased vulnerability. While critical to mitigating the spread of COVID-19, many experts were concerned about the potential impacts of these measures on the mental health of older adults (Pfefferbaum & North, 2020). Social isolation among this population was already considered a public health crisis prior to the COVID-19 pandemic (Berg-Weger & Morley, 2020; Cacioppo & Cacioppo, 2018) and, given the strong associations of social isolation with mental health outcomes such as depression, anxiety and suicidal ideation (Calati et al., 2019; Ong et al., 2016; Santini et al., 2020), the mental health consequences were expected to be significant.

In response to this emerging context, we developed a photovoice study investigating social isolation and mental health of older adults during the pandemic. This project was motivated by our interest and expertise in mental health promotion and older adults’ health, as well as our team prior successes implementing photovoice method (Ferlatte et al., 2019; Glenn et al., 2020). We chose photovoice for the following reasons. First, photovoice is an effective and unique strategy to study mental health as it provides a mechanism for participants to access, reflect upon and authentically reveal their experiences (Han & Oliffe, 2016). Second, exhibits of photovoice photographs can raise awareness about issues that are under-reported or constructed in ways that stigmatize people (Thompson et al., 2008). Therefore, with the participant’s consent, we would be able to develop an online exhibit of the photographs generated during the project to stimulate dialogue about the issues of social isolation and mental health during the COVID-19 pandemic. Third, as social isolation of older adults was emerging as a significant concern in discourses about the pandemic, a photovoice study could be an intervention in itself (Ferlatte & Oliffe, 2019a; Sitvast et al., 2010), creating a safe space for older adults to discuss and share their experiences in productive ways. Our detailed approach is described in the method section below.

## Purpose

The intent of this article is to discuss the implementation of a virtual photovoice study and present some of the challenges of this study design and potential solutions from our perspectives as researchers. Inductively derived through team discussions, reflexive memos and the writing of the current article, we share our collective insights into conducting a virtual photovoice with older adults. To our knowledge, this project is the first photovoice study with older adults to collect data completely virtually. While our results are specific to older adults, our insights may be useful to other study populations.

## Method

### Recruitment of Participants

We recruited a sample of 26 older adults to participate in our photovoice project between May and November 2020. Participants were eligible if they were 60 years of age or older, spoke and understood French, and resided in the province of Quebec. As participation in the photovoice activities were completely online, participants were required to have an internet connection as well as a phone, tablet, or digital camera they could use to take photographs. The study included three sub-groups of older adults: older adults living in retirement homes, older adults living alone, and older adults identifying as members of the lesbian, gay, bisexual and transgender community (LGBTQ). These sub-groups were identified in collaboration with community partners and were selected because they were identified as vulnerable to social isolation and to the negative mental health impacts of COVID-19. As such, we believed that individuals within these sub-groups would particularly benefit from participating in the study and of discussing their experience of the pandemic with peers. By focusing on groups of older adults that are particularly at risk of experiencing negative outcomes and by providing a safe space for them to meet peers and exchange their experience of confinement, our study aligns with the aim of photovoice method which is to empower communities. Participants were recruited through retirement homes and community groups who shared an invitation directly to their member in person, in their newsletters or on social media. The majority of participants were female (*n* = 21), white (*n* = 25) and the mean age was 71 years old (range 60 to 81).

### Procedures

Participation in the project occurred in several steps. Participants first met online one-on-one with the study coordinator for an intake interview. The purpose of this meeting was to explain the study and the photovoice component, obtain consent, collect basic demographic information, and provide any additional details to participants about the project. This meeting also helped to build rapport between the participants and the research coordinator. Participants were then invited, over a period of 3 weeks, to take a series of photographs to tell their story of the pandemic and confinement, with a focus on the mental health impacts and their mitigation strategies.

**Figure 1.**
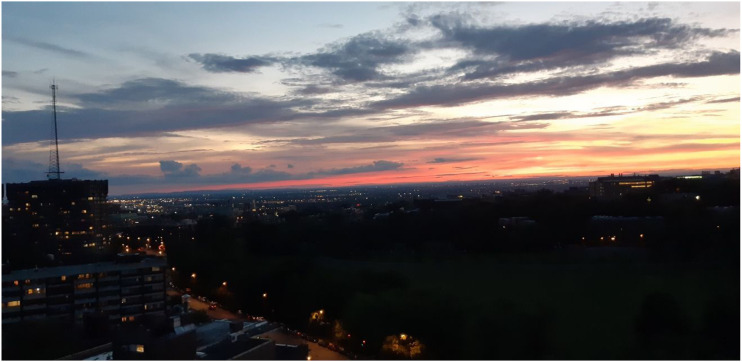
“Sunset from my balcony. I am comforted admiring this colorful spectacle”.

**Figure 2.**
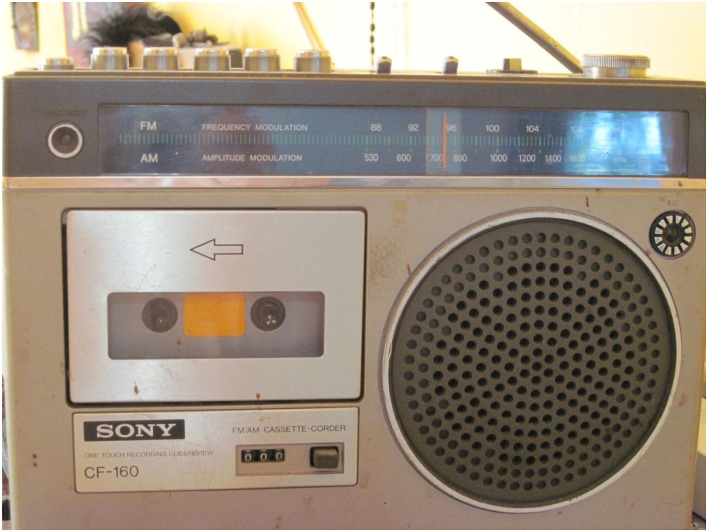
“Information about the virus broadcast to my radio that is on 24 hours a day”.

**Figure 3.**
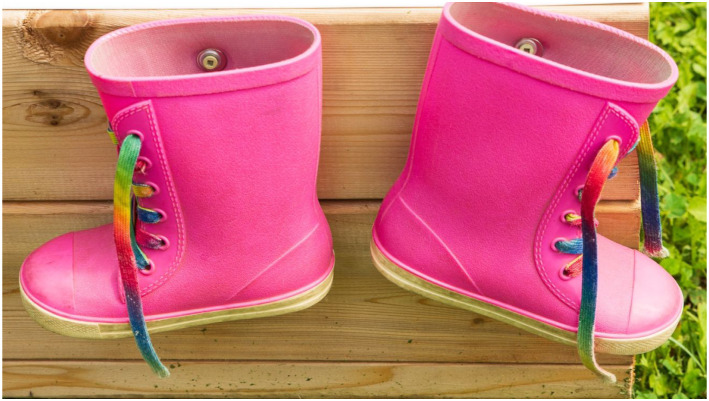
“Life continues and renews itself, we must nourish it with beauty and small pleasures, this will make us forget our misfortunes”.

**Figure 4.**
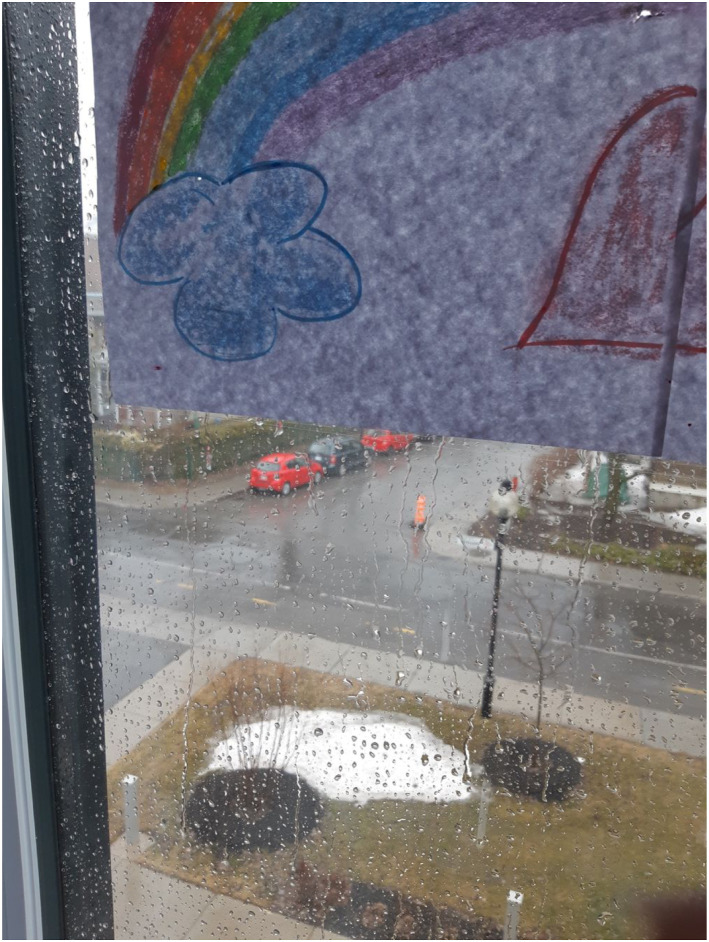
“The rain streams down my rainbow. Time passes, sometimes gloomy and sad. From my bedroom window, I can’t see the busyness of everyday life anymore. But it’s going to be fine!”.

Each week during the photovoice assignment, participants participated in an online group to discuss their photographs with others. These discussion groups lasted approximately 90 minutes and involved between 5 to 7 participants. The sessions were facilitated by the principal investigator and a doctoral student trained in qualitative research. Each session began with participants describing their week and their experiences taking photographs. Then, each participant was invited to present and describe her/his photographs. After this the other participants were invited to comment or share whether they had similar or divergent experiences or perspectives. Participants received CAD $60 for their participation. Detailed notes were taken during the discussion groups by the principal investigators. When data collection was completed, selected participant-produced photographs that represented a breath of experiences and perspectives were chosen by the research team to create an online exhibit to raise public awareness about the experience of older adults during the COVID-19 pandemic (https://confinesensemble.ca/). A selection of photographs from our participants is available in Figures 1–4 as examples and as evidence of the feasibility of conducting a virtual photovoice study with older adults.

### Ethics

As per Canadian policies and standards with regards to conducting research involving humans, our project was submitted, reviewed, and approved by the Université de Montréal ethics review board (*Comité d’éthique de la recherche en sciences et en santé*). As we launched our study in the first few months of the pandemic, it was impossible to visit participants to obtain their written consent as in-person research activities were strictly forbidden by our institution. Demanding an online signature could have constituted a barrier to participation for older adults who are less familiar with technologies and may not have a printer or an online signature, as such we requested and obtained a permission from our ethics board to obtained consent orally. Individuals who expressed interest to participate in the study received by email a document containing all the information to allow them to make an informed decision about their participation. They were given at least 24 hours before a phone or online meeting (the participant’s preference) was set up to obtain their consent, which was noted in our records. Participants first provided consent to participate in the study and then to the use of their photographs for dissemination. Consent for the dissemination of the photographs was obtained after they had time to consider when and where they wanted their photographs shared. Participants were invited to reflect on the potential harms to have their photographs available, particularly online where they can easily be accessed and reproduced. Participants could opt to have their photographs not shared outside of the research team, which none of the participants did. The participants also indicated how they would be identified as the authors/artists of their photographs, either by their first name or a pseudonym of their liking. Participants were informed that they could withdraw their consent to the use of the photographs at any time by contacting the research team. Considering the anxiogenic context in which we conducted our study, a comprehensive list of mental health resources – including resources specific to older adults - was provided to the participants. Participants were reminded of the list at each meeting and were invited to connect with the research team if they needed assistance in connecting with the resources listed.

### Challenges and Reflections

Over the course of the study, the first two authors (OF and JK), who conducted the data collection, held debriefing sessions immediately after each discussion group meeting. These debriefs offered an opportunity to discuss emerging issues and exchange observations on the functioning of the discussion groups and the overall photovoice project. The first author took detailed notes of these discussions and summarized key discussion points including emerging reflections and ideas. The particularly salient and complex issues that the first two authors felt warranted further discussions and reflection were then addressed in the larger research group meetings. These meetings helped identify opportunities to improve data collection and the experience of the participants, as well as reflect on the use of virtual photovoice research design with older adults. Using notes from our discussions and reflexive memos, we identified three main challenges that we needed to address during our project.

## Results

We experienced three main challenges in the implementation of our virtual photovoice study: managing technical difficulties, fostering a positive group dynamic, and building and maintaining trust. The following section explains each challenge and how we adapted.

### Managing Technical Difficulties

As the study was conducted completely online, participants were required to have internet access and a means to take and send digital photographs. Participants in the study were somewhat required to be familiar and competent with technologies. We, nonetheless, faced a few technical challenges. First, some participants faced difficulties in mastering the online meeting platform. Our study used *Zoom*, which was the platform recommended by our research ethics board for online data collection due to its many security features and the ability to record meetings directly on a password protected computer. While some participants had used online meeting planforms before, many were new to this technology. The intake interview was conducted over the phone or online, depending on the level of comfort of the participant with the technology. During this first meeting, we spent significant time setting up each participant for the online discussion groups, including assisting with creating accounts, downloading the application and providing a tutorial of the basic meeting room functions. As we were unable to visit participants due to strict restrictions related to COVID-19, this support was done over the phone, which created some difficulties as we did not have a visual of what their participants were doing on their devices. Our participants used a variety of devices to connect to our meetings such as computers, tablets and smartphones*.* As the functions vary across these devices, our research team needed to be familiar with all of them to provide support. For example, the most common difficulty experienced by participants was activating the video function. This function is on opposite corners of the user interface on a tablet when compared with a computer. Being familiar with the meeting platform functionalities on various devices, and keeping a log of each participants’ device, allowed us to quickly work through technical challenges during the meetings.

Despite our efforts to ensure the participants were familiar with the meeting platform ahead of the group discussions, many participants experienced difficulties in joining the meetings. It was important to have two researchers present at each discussion group so that one researcher could call and assist participants over the phone without delaying the start of the meeting. It became apparent that participants were often unable to connect because they were using a previous or wrong link, we created a recurrent meeting room that used the same link and sent the link to participants on the day of the meeting, to make the correct meeting link easier to access. Aside from the meeting platform, many participants were unfamiliar with sending photographs via email, as requested in our instructions. Instead of teaching them how to send photographs via email, we amended our protocol to accept the photographs via *Messenger* or text, which participants were often more familiar with.

Finally, we faced issues related to the quality of the audio recording during the discussion groups. Online meeting technologies such as *Zoom* have automatic volume adjustment that decreases the sound of the person speaking when other noises are present (e.g., other participants and researchers’ reactions, laugh, ambient noise). This led to many inaudible fragments in our audio recording, challenging accurate transcriptions. To improve the quality of the audio recording, our transcriber suggested we ask participants (and the researchers) to wear headsets and mute their microphones when not speaking. Participants who had headsets were encouraged to wear them. However, after discussion with the study team, we decided not to ask participants to mute themselves, because of concerns that it would negatively affect the group dynamic and add a layer of difficulty for the participants who were already struggling with the platform. To avoid interference, the discussion facilitator made efforts to reduce empathy marks and encouraging reactions (such as *hum*, *ok*, etc.) during the participants’ narratives. Detailed notes were taken at the meetings as a backup for segments missed during the recording.

### Fostering a Positive Online Group Dynamic

As pointed out by others (Reisner et al., 2018), fostering a positive online group dynamic and a sense of belonging and connection online can be difficult without in-person contact, particularly for older adults who may have less experience with this form of communication. Therefore, conscious efforts and adjustments throughout the project were made to foster a positive and intimate experience for the participants. First, we adjusted the size of our discussion groups. Wang and Burris (1997) originally proposed a sample size of six to ten participants for photovoice studies, consistent with the focus group literature that recommends seven to ten participants (Morgan, 2012). We originally planned for ten participants per discussion group and our first group had seven participants. However, this first discussion felt chaotic and was difficult to manage at times, with some participants trying to speak on top of one-another and others withdrawing or becoming distracted (e.g., doing other tasks during the meeting). To overcome this issue, we reconfigured the group discussion to a maximum of five participants and two researchers, which resulted in a more intimate ambiance which facilitated interactions.

The two researchers that facilitated the group discussions were in their 30s and 40s and as such were “outsiders”. Yet, we believe this position motivated the participants to discuss their stories as they took the task of educating us about how they experienced and managed the pandemic and its social distancing measures. During the project, participants often voiced that they felt stigmatized as older adults by public health messages and that the media poorly represented their experiences. This resulted in them feeling invisible. As such, participants often expressed gratitude that younger researchers took the time to listen to their stories and concerns. Because the conversations were centered on the participants’ produced photographs, the topics discussed reflected their priorities and facilitated a dynamic where the researchers were simply there to learn from the participants rather than interrogate and lead the discussion.

Time management was an issue we experienced that threatened the group dynamics. As participants were already spending a significant amount of time on the project, making sure that the online group discussion did not go over time, and lead to “*Zoom fatigue*”, was a priority. Reducing the number of participants per group made it easier to keep the meetings to under 90 minutes. We also revised the number of photographs to be submitted by participants. We initially asked participants to submit four to five photographs, but this was too many to keep meetings under 90 minutes, and sometimes led to long descriptions of the photographs with little interaction between group members. We therefore reduced the number of photographs to three, which resulted in more lively and enjoyable sessions. The photographs were also compiled and organized into a PowerPoint presentation ahead of time to ease the flow of the meeting. The presentation was displayed by a researcher using the share screen option.

In terms of presenting the photographs to the group, we initially envisioned that participants would present their photos one by one, soliciting feedback and thoughts from other participants after each photograph. This resulted in many themes being repeated over the course of a single session. Discussions went off topic when participants submitted photos unrelated to the project’s subject. We also sensed early on that this format created a certain pressure on participants for each photo to be a revelation. To create a more dynamic discussion and reduce performance anxiety, we quickly moved to having each participant present their photographs all at once and then opened up the group discussion. We also further focused the exchanges by suggesting a discussion theme based on the photographs or narrative of the presenting participant. This approach led to more focused conversations on themes that were inclusive, diverse and interesting, and balanced discussions that covered both negative aspects related to the pandemic (e.g., solitude, anxiety, anger) and positive aspects (e.g., resilience of older adults, social support). This need for a more balanced discussion was prompted after a participant withdrew from the project citing the negative tone of the session.

### Building and Maintaining Trust

The building of trust and caring relationships are key to the good functioning of a photovoice study (Call-Cummings et al., 2019). Yet, building trust can be challenging online, particularly with older adults who may be less comfortable introducing themselves and communicating virtually. To work successfully, we felt that participants had to trust us as researchers as much as we needed the participants to trust one another so that they would be open to sharing their experiences, thoughts and perspectives. Building trust takes time. As such, during the intake interviews, the interviewer took the time to get to know the participant and to share a little bit about herself and her own experience of the pandemic. The researchers also kept notes of what was shared by the participants so the details could be referenced at a later date. For example, if a participant mentioned her birthday was coming in a few days, or that a child was planning a visit, the interviewer would then enquire about these events at a subsequent meeting. This was a simple but effective way to demonstrate that the researchers cared about the participants. Moreover, we elected that the person conducting the intake interview would also complete the follow-ups with the participants as well as lead the discussion groups to maintain the relationship of trust. We realized early on that the presence of a consistent and reliable researcher throughout the project was important for participants who often inquired who would be present at the group discussions during the intake interview.

It was also important that participants had trust in each other. Therefore, at the first meeting, we allowed a considerable amount of time (15 to 20 minutes) for participants to introduce themselves. We began each subsequent meeting by having each participant describe their week before starting the formal photovoice discussion. We also reminded participants at each meeting about the confidentiality agreement to boost trust. While we had originally intended for participants to attend a single group session, we eventually opted for three discussion groups after discussing with our community partners, as a means to create a safe space for participants who might be reluctant to share personal information or their emotions with others they had never met. Holding multiple sessions also aligned with our desire to have this project reduce social isolation. The value of multiple sessions was evident in our study as participants usually progressed from sharing daily inconveniences of the pandemic during the first session (e.g., difficulty obtaining groceries, not being able to go to the gym) to more intimate and emotional impacts during the last session (e.g., feelings of anxiety, loneliness, fear and uncertainties for the future).

Trust can be broken easily, particularly in an anxiety-provoking context such as the COVID-19 pandemic. As such, we paid careful attention to the participants’ behaviours during the meeting, which was facilitated by using the gallery view function on the online meeting platform during the discussions. Debriefing session between the researchers who collected the data usually started by sharing their impression of each participant. If we noticed a participant seemed irritated or distant, we often followed up with this person by phone or email. While we initially worried that this effort would be perceived as invasive or dismissed by participants, it rather provided an opportunity for participants to share their concerns, which were later addressed. For example, we noticed in one meeting that a participant withdrew himself and stopped engaging halfway through. When we followed up with him, he mentioned that he was considering leaving the study because he was irritated by another participant who often talked about unrelated topics to the study and provided a photo that he perceived as mocking the Catholic Church. We then followed up with the other participant to encourage him to stay on topic. This interaction helped restore the trust in the group, leading to both participants more authentically sharing their experiences.

## Discussion

The process of planning and implementing a completely virtual study with older adults required careful consideration and adaptation related to the use of technology, fostering positive group dynamics, and building trust. A common thread for finding solutions in this context were patience and time. Photovoice is already a method that is labor-intensive and time-consuming. Conducting all phases of a photovoice project virtually with older adults who had varying degrees of familiarity with online technologies added a layer of complexity and increased the length of many activities. Older adults are increasingly using technology in their everyday lives but they remain more likely to feel anxiety with the use of new technologies (Nimrod, 2018; Wang & Chen, 2015). We witnessed varying degree of anxiety with the technological aspects of our study among our participants. We often had to connect with them outside of formal study meetings to assist with technical issues such as connecting to the online meeting platform, sharing photographs virtually, or assisting with photograph selection. These additional meetings could last anywhere from a few minutes to an hour and required the researcher be available to respond quickly. These meetings were very helpful in building the participants’ confidence with the tool used during the study and for them to acquire new skills with technology, therefore meeting in some manner the aim of photovoice to empower communities.

There are several lessons to be learned from our experience regarding the planning and implementation of virtual photovoice studies. The learning curve related to online meetings can be steep for some participants who have less experience with technology. Researchers therefore need to plan and build in the time and resources for technological support before and outside of photovoice meetings. We encourage researchers using online photovoice to pay particular attention to issues related to group dynamics and trust. Organizing the photos carefully into a presentation was found to be particularly useful and ensured the meetings were engaging and ran smoothly. In the context of older adults, it provided a structure that was appreciated by the participants while reducing the anxiety of the participants of sharing the photographs. Key to building trust with our study population was having one person as the main point of contact outside of the meetings but that also facilitated the discussion. This made it easier for them to reach out to discuss their concerns with the study and ask for help. Finally, we suggest researchers have regular debriefing sessions to discuss emerging reflections and issues and use methodological memos to provide a mechanism to keep a record of these issues and offer an opportunity to reflect on potential solutions as well as to engage community partners.

While we detailed some of our observations and reflections, the implementation of our virtual photovoice was not subjected to a formal evaluation. We recommend that formal evaluation mechanisms be integrated in future virtual photovoice studies to better capture the point of view of participants on this method. The present study demonstrates the feasibility of doing a photovoice using synchronous online discussion groups. However, one of the advantages of online research is that it allows asynchronous data collection. Although other researchers have done asynchronous photovoice with youth and adults (Lichty et al., 2019; Tanhan & Strack, 2020), the acceptability of this form of photovoice remains unknown among older adults who may be less familiar or less motivated to post photographs and comment on others’ photos without live interactions.

### Limitations

The current article does not cover all methodological challenges of conducting a photovoice study virtually. Online studies can be subject to complex ethical issues related to privacy and authenticity (James & Busher, 2007) that have yet to be examined in the context of photovoice and that are beyond the scope of the present paper. Our article does not address some of the potential biases and exclusion issues arising as a result of using an online medium and therefore does not discuss “whose voice” was represented in our study. While older adults are increasingly using the internet, inequities in terms of class, education and dis/ability continue to fuel a growing digital divide among older adults (Hunsaker & Hargittai, 2018). In the context of our research, we are deeply aware that older adults who are the most at risk of social isolation and mental illness during the pandemic might be the ones lacking internet access, and therefore were not included in our study. We did not collect information on ability/disability in our study and none of the participants disclosed a disability within the course of the project. Yet, 37% of older adults live with a disability (Statistics Canada, 2018). Future work should explore the challenges and opportunities to conduct photovoice online with this sub-population of older adults. It is plausible that conducting discussion groups online can facilitate participation of those living with a disability, particularly for those with hearing difficulties, as each participant can adjust their own volume and online meeting platforms offer the possibility for live captioning. Individuals with mobility issues can also take part in the research without having to leave their house. Overall, more work is needed theoretically, methodologically and empirically to highlight the ethical challenges specific to virtual photovoice study and to identify effective strategies to include the most vulnerable in this research approach, including those with disabilities.

## Conclusion

In a growing digital world, conducting qualitative research online is increasingly popular (Woodyatt et al., 2016). In the context of the COVID-19 pandemic and social distancing measures, online research was no longer an option but a necessity (Vindrola-Padros et al., 2020). Yet, online research can be a challenge for methods that are grounded in participatory research principles and that rely heavily on face-to-face interactions between participants and researchers. Nonetheless, with careful planning, flexibility, commitment and ongoing reflection, conducting a completely virtual photovoice is not only feasible, but a novel way to connect with a wide range of participants and to move participatory research forward into the future.

## References

[bibr1-16094069221095656] Baker T. A. Wang C. C. (2006). Photovoice: Use of a participatory action research method to explore the chronic pain experience in older adults. Qualitative Health Research, 16(10), 1405–1413. 10.1177/1049732306294118 17079801

[bibr2-16094069221095656] Berg-Weger M. Morley J. E. (2020). Loneliness in old age: An unaddressed health problem. Journal of Nutrition, Health and Aging, 24(3), 243–245. 10.1007/s12603-020-1323-6 PMC7223173 32115602

[bibr3-16094069221095656] Bryanton O. Weeks L. Townsend E. Montelpare W. Lees J. Moffatt L. (2019). The utilization and adaption of photovoice with rural women aged 85 and older. International Journal of Qualitative Methods, 18, 1–9. 10.1177/1609406919883450

[bibr4-16094069221095656] Budig K. Diez J. Conde P. Sastre M. Hernán M. Franco M. (2018). Photovoice and empowerment: Evaluating the transformative potential of a participatory action research project. BMC Public Health, 18(1), 432. 10.1186/s12889-018-5335-7 29609576 PMC5879794

[bibr5-16094069221095656] Cacioppo J. T. Cacioppo S. (2018). The growing problem of loneliness. The Lancet, 391(10119), 426. 10.1016/S0140-6736(18)30142-9 PMC6530780 29407030

[bibr6-16094069221095656] Calati R. Ferrari C. Brittner M. Oasi O. Olié E. Carvalho A. F. Courtet P. (2019). Suicidal thoughts and behaviors and social isolation: A narrative review of the literature. Journal of Affective Disorders, 245, 653–667. 10.1016/j.jad.2018.11.022 30445391

[bibr7-16094069221095656] Call-Cummings M. Hauber-Özer M. Byers C. Mancuso G. P. (2019). The power of/in Photovoice. International Journal of Research and Method in Education, 42(4), 399–413. 10.1080/1743727X.2018.1492536

[bibr8-16094069221095656] Catalani C. Minkler M. (2010). Photovoice: A review of the literature in health and public health. Health Education and Behavior, 37(3), 424–451. 10.1177/1090198109342084 19797541

[bibr9-16094069221095656] Duffy L. (2011). “Step-by-Step we are stronger”: Women’s empowerment through photovoice. Journal of Community Health Nursing, 28(2), 105–116. 10.1080/07370016.2011.564070 21541872

[bibr10-16094069221095656] Ferlatte O. Oliffe J. (2019a). Lobbying suicide prevention policy for gay and bisexual Men: An intersectionality-informed photovoice project. In The palgrave handbook of intersectionality in public policy (263–284). Palgrave MacMillan. 10.1007/978-3-319-98473-5

[bibr11-16094069221095656] Ferlatte O. Oliffe J. L. (2019b). Picturing intersectionality in men’s suicidality research. In Griffith D. Bruce M. A. Thorpe R. J. (Eds.), Men’s health equity (pp. 524–540). 10.4324/9781315167428-33

[bibr12-16094069221095656] Ferlatte O. Oliffe J. L. Salway T. Broom A. Bungay V. Rice S. (2019). Using photovoice to understand suicidality among gay, bisexual, and two-spirit men. Archives of Sexual Behavior, 48(5), 1529–1541. 10.1007/s10508-019-1433-6 31152366

[bibr13-16094069221095656] Fielding N. G. Lee R. M. Blank G. (2016). The SAGE handbook of online research methods. Sage. 10.4135/9781473957992

[bibr14-16094069221095656] Fitzpatrick A. L. Steinman L. E. Tu S. P. Ly K. A. Ton T. G. N. Yip M. P. Sin M. K. (2012). Using photovoice to understand cardiovascular health awareness in Asian elders. Health Promotion Practice, 13(1), 48–54. 10.1177/1524839910364381 21057047 PMC11753058

[bibr15-16094069221095656] Glenn N. M. Frohlich K. L. Vallée J. (2020). Socio-spatial inequalities in smoking among young adults: What a ‘go-along’ study says about local smoking practices. Social Science and Medicine, 253, 112920. 10.1016/j.socscimed.2020.112920 32240888

[bibr16-16094069221095656] Golden T. (2020). Reframing photovoice: Building on the method to develop more equitable and responsive research practices. Qualitative Health Research, 30(6), 960–972. 10.1177/1049732320905564 32081060

[bibr17-16094069221095656] Han C. S. Oliffe J. L. (2016). Photovoice in mental illness research: A review and recommendations. Health: An Interdisciplinary Journal for the Social Study of Health, Illness and Medicine, 20(2), 110–126. 10.1177/1363459314567790 PMC4768711 25673051

[bibr18-16094069221095656] Hergenrather K. C. Rhodes S. D. Cowan C. A. Bardhoshi G. Pula S. (2009). Photovoice as community-based participatory research: A qualitative review. American Journal of Health Behavior, 33(6), 686–698. 10.5993/AJHB.33.6.6 19320617

[bibr19-16094069221095656] Hunsaker A. Hargittai E. (2018). A review of Internet use among older adults. New Media and Society, 20(10), 3937–3954. 10.1177/1461444818787348

[bibr20-16094069221095656] James N. Busher H. (2007). Ethical issues in online educational research: Protecting privacy, establishing authenticity in email interviewing. International Journal of Research and Method in Education, 30(1), 101–113. 10.1080/17437270701207868

[bibr21-16094069221095656] Janghorban R. Roudsari R. L. Taghipour A. (2014). Skype interviewing: The new generation of online synchronous interview in qualitative research. International Journal of Qualitative Studies on Health and Well-Being, 9(1), 24152. 10.3402/qhw.v9.24152 24746247 PMC3991833

[bibr22-16094069221095656] Lichty L. Kornbluh M. Mortensen J. Foster-Fishman P. (2019). Claiming online space for empowering methods: Taking photovoice to scale online. Global Journal of Community Psychology Practice, 10(3), 1–26. https://www.gjcpp.org/en/article.php?issue=33&article=201

[bibr23-16094069221095656] Liebenberg L. (2018). Thinking critically about photovoice: Achieving empowerment and social change. International Journal of Qualitative Methods, 17, 160940691875763. 10.1177/1609406918757631

[bibr24-16094069221095656] Mahmood A. Chaudhury H. Michael Y. L. Campo M. Hay K. Sarte A. (2012). A photovoice documentation of the role of neighborhood physical and social environments in older adults’ physical activity in two metropolitan areas in North America. Social Science and Medicine, 74(8), 1180–1192. 10.1016/j.socscimed.2011.12.039 22365935 PMC10339377

[bibr25-16094069221095656] Morgan D. (2012). Focus groups as qualitative research. Sage. 10.4135/9781412984287

[bibr26-16094069221095656] Nimrod G. (2018). Technophobia among older Internet users. Educational Gerontology, 44(2–3), 148–162. 10.1080/03601277.2018.1428145

[bibr27-16094069221095656] Novek S. Menec V. H. (2014). Older adults’ perceptions of age-friendly communities in Canada: A photovoice study. Ageing and Society, 34(6), 1052–1072. 10.1017/S0144686X1200150X

[bibr28-16094069221095656] Novek S. Morris-Oswald T. Menec V. (2012). Using photovoice with older adults: Some methodological strengths and issues. Ageing and Society, 32(3), 451–470. 10.1017/S0144686X11000377

[bibr29-16094069221095656] Oliffe J. L. Bottorff J. L. (2007). Further than the eye can see? Photo elicitation and research with men. Qualitative Health Research. 10.1177/1049732306298756 17582026

[bibr30-16094069221095656] Ong A. D. Uchino B. N. Wethington E. (2016). Loneliness and health in older adults: A mini-review and synthesis. Gerontology, 62(4), 443–449. 10.1159/000441651 26539997 PMC6162046

[bibr31-16094069221095656] Panazzola P. Leipert B. (2013). Exploring mental health issues of rural senior women residing in southwestern Ontario, Canada: A secondary analysis photovoice study. Rural and Remote Health, 13(2), 2320. 10.22605/RRH2320 23781863

[bibr32-16094069221095656] Pfefferbaum B. North C. S. (2020). Mental health and the covid-19 pandemic. New England Journal of Medicine. 10.1056/nejmp2008017 32283003

[bibr33-16094069221095656] Reisner S. L. Randazzo R. K. White Hughto J. M. Peitzmeier S. DuBois L. Z. Pardee D. J. Marrow E. McLean S. Potter J. (2018). Sensitive health topics with underserved patient populations: Methodological considerations for online focus group discussions. Qualitative Health Research, 28(10), 1658–1673. 10.1177/1049732317705355 29298574 PMC5758419

[bibr34-16094069221095656] Santini Z. I. Jose P. E. York Cornwell E. Koyanagi A. Nielsen L. Hinrichsen C. Meilstrup C. Madsen K. R. Koushede V. (2020). Social disconnectedness, perceived isolation, and symptoms of depression and anxiety among older Americans (NSHAP): A longitudinal mediation analysis. The Lancet Public Health, 5(1), e62–e70. 10.1016/S2468-2667(19)30230-0 31910981

[bibr35-16094069221095656] Sitvast J. E. Abma T. A. Widdershoven Guy G. A. M. (2010). Facades of suffering: Clients’ photo stories about mental illness. Archives of Psychiatric Nursing, 24(5), 349–361. 10.1016/j.apnu.2010.02.004 20851326

[bibr36-16094069221095656] Statistics Canada. (2018). A demographic, employment and income profile of Canadians with disabilities aged 15 years and over, 2017. https://www.ldac-acta.ca/canadian-survey-on-disability-reports-a-demographic-employment-and-income-profile-of-canadians-with-disabilities-aged-15-years-and-over-2017/

[bibr37-16094069221095656] Strack R. W. Magill C. McDonagh K. (2004). Engaging youth through photovoice. Health Promotion Practice, 5(1), 49–58. 10.1177/1524839903258015 14965435

[bibr38-16094069221095656] Tanhan A. Strack R. W. (2020). Online photovoice to explore and advocate for Muslim biopsychosocial spiritual wellbeing and issues: Ecological systems theory and ally development. Current Psychology, 39(6), 2010–2025. 10.1007/s12144-020-00692-6

[bibr39-16094069221095656] Thompson N. C. Hunter E. E. Murray L. Ninci L. Rolfs E. M. Pallikkathayil L. (2008). The experience of living with chronic mental illness: A photovoice study. Perspectives in Psychiatric Care, 44(1), 14–24. 10.1111/j.1744-6163.2008.00143.x 18177274

[bibr40-16094069221095656] Vindrola-Padros C. Chisnall G. Cooper S. Dowrick A. Djellouli N. Symmons S. M. Martin S. Singleton G. Vanderslott S. Vera N. Johnson G. A. (2020). Carrying out rapid qualitative research during a pandemic: Emerging lessons from COVID-19. Qualitative Health Research, 30(14), 2192–2204. 10.1177/1049732320951526 32865149 PMC7649912

[bibr41-16094069221095656] Wang C. Burris M. A. (1997). Photovoice: Concept, methodology, and use for participatory needs assessment. Health Education & Behavior, 24(3), 369–387. 10.1177/109019819702400309 9158980

[bibr42-16094069221095656] Wang C. C. Chen J. J. (2015). Overcoming technophobia in poorly-educated elderly - the HELPS-seniors service learning program. International Journal of Automation and Smart Technology, 5(3), 173–182. 10.5875/ausmt.v5i3.980

[bibr43-16094069221095656] Woodyatt C. R. Finneran C. A. Stephenson R. (2016). In-person versus online focus group discussions: A comparative analysis of data quality. Qualitative Health Research, 26(6), 741–749. 10.1177/1049732316631510 26935719

[bibr44-16094069221095656] Yankeelov P. A. Faul A. C. D’Ambrosio J. G. Collins W. L. Gordon B. (2015). “Another day in paradise”: A photovoice journey of rural older adults living with diabetes. Journal of Applied Gerontology, 34(2), 199–218. 10.1177/0733464813493136 24652892

